# Dental Recorder

**Published:** 1852-01

**Authors:** 


Dental Recorder.—We welcome to the editorial corps Dr. Hill, who has recently become associated with Dr. Allen in the editorship of the Recorder. Dr. Hill is a good writer, and we believe much devoted to the profession.

The Recorder, we should think, has a pretty extensive circulation, and from the many enquiries made for it recently, we judge it is very much appreciated in this quarter.

We would, however, take this opportunity of saying to the editors, that the last Nos. have not been received in this city. We believe the last received was the October number. Is this the last issued ?

